# Clinicians’ perspectives on complementary and alternative medicine use in headache care across Africa and West Asia: a cross-sectional survey

**DOI:** 10.3389/fneur.2026.1849417

**Published:** 2026-10-13

**Authors:** Amr Hassan, Freda Dodd-Glover, Rehab Magdy, Aynur Ӧzge, Derya Uludüz, Najib Kissani, Ahmed Allioueche, Aussan Al-Athwari, Emna Ellouz, Sounga Bandzouzi Prince Eliot Galieni, Mendinatou Agbetou, Kigocha Okeng'o, Daniel Gams Massi, Evelyne Diarra, Agbo Panzo Cedric, Chiamaka Edith Okereke, Wael Alwachi, Osheik Seidi, Kadira Abdi Aden, Augustina Charway-Felli, Foksouna Sakadi, John N. Jabang, Denis R. Shatima, Athanase Millogo, Magnerou Annick Melanie, Fatimata Hassane Djibo, Tiwonge Elisa Phiri, Jamal Barros Baco, Grenaba-Duval Lewis Reinier Joël, Frighton Mutete, Jasem Alhashel, Mansoureh Togha, Ammar Fayez Mubaidin, Sarkhan Amirguliyev, Abderrahmane Moulaye, Hana Kedah, Aya Alsayyad, Doaa M. khalil, Mona Hussein

**Affiliations:** 1 Department of Neurology, Kasr Alainy Faculty of Medicine, Cairo University, Cairo, Egypt; 2 Neurology Unit, Department of Medicine and Therapeutics, Korle Bu Teaching Hospital, Accra, Ghana; 3 Department of Neurology, Headache Clinic, Mersin, Türkiye; 4 NOROM Neuroscience and Excellence Center, Ankara, Türkiye; 5 Neurologist Headache Specialist, Board Member of International Headache Society, Department of Neurology, Faculty of Medicine, Istanbul Atlas University, Istanbul, Türkiye; 6 Headache and Epilepsy Clinic, Past Head of Neurology and Neuroscience Department, Mohamed 6th University Hospital, Marrakech, Morocco; 7 Department of Neurology, Um6p University, Bengrir, Morocco; 8 CHU Constantine, Private Neurologist, Constantine, Algeria; 9 Consultant Neurologist and Neuro-interventionist, Aden University, Aden, Yemen; 10 Department of Neurology, Gabes Hospital, Sfax University (Université de Sfax), Sfax, Tunisia; 11 Department of Neurology, Loandjili General Hospital, Pointe-Noire, Republic of Congo; 12 Faculty of Health Sciences, Marien Ngouabi University, Brazzaville, Republic of Congo; 13 Department of Neurology, University of Parakou, Parakou, Benin; 14 Muhimbili National Hospital, Dar es Salaam, Tanzania; 15 Faculty of Medicine and Pharmaceutical Sciences, University of Doula, Douala, Cameroon; 16 Department of Neurology, CHU Cocody, University Felix Houphouet, Boigny-Abidjan, Côte d'Ivoire; 17 University of Nigeria Teaching Hospital, Enugu, Nigeria; 18 Neurology Specialist, Head of Neurology Department at Ali Omar Askar Hospital, Tripoli, Libya; 19 Faculty of Medicine, University of Khartoum, Khartoum, Sudan; 20 Hôpital Militaire de Djibouti, Djibouti City, Djibouti; 21 37 Military Hospital, Accra, Ghana; 22 Department of Neurology, Nationale Reference Teaching Hospital, N'Djamena, Chad; 23 Edward Francis Small Teaching Hospital, University of Gambia, Serekunda, Gambia; 24 Department of Paediatrics, National Hospital Abuja, Abuja, Nigeria; 25 Centre Hospitalier Universitaire, Souro Sanou, Burkina Faso; 26 Faculty of Medicine and Pharmaceutical Sciences, University of Douala, Douala, Cameroon; 27 Department of Neurology, Hospital National Amirou Boubacar Diallo of Niamey, Niamey, Niger; 28 Queen Elizabeth Central Hospital, Blantyre, Malawi; 29 Hospital Central da Beira, Beira, Mozambique; 30 Department of Neurology, Université de Bangui, Bangui, République Centrafricaine; 31 Livingstone University Teaching Hospital, Livingstone, Zambia; 32 Department of Neurology, Kuwait University, Kuwait City, Kuwait; 33 Department of Neurology and Headache Fellowship, Headache Department, Iranian Center of Neurological Research, Neuroscience Institute, Tehran University of Medical Sciences, Tehran, Iran; 34 Headache Department, Neurology Ward, Sina Hospital, Medical School, Tehran University of Medical Sciences, Tehran, Iran; 35 Khalidi Medical Center, Amman, Jordan; 36 Migraine and Headache Specialist, Baku, Azerbaijan; 37 Department of Neurologist, Nouakchott, Mauritania; 38 Neurology Consultant, Head of Neurology Department and Program Director of Neurology Saudi Board at Hear Hospital, Makkah, Saudi Arabia; 39 Neurology Consultant, MOH, Sharjah, United Arab Emirates; 40 Public Health and Community Medicine, Beni-Suef University, Beni Suef, Egypt; 41 Department of Neurology, Beni-Suef University, Beni-Suef, Egypt

**Keywords:** Africa, complementary and alternative medicine, headache disorders, low-income countries, West Asia

## Abstract

**Background:**

Headache disorders impose a substantial burden across Africa and West Asia, where limited access to healthcare may increase reliance on complementary and alternative medicine (CAM). The current study explored clinicians’ perspectives on CAM use, safety concerns, and research needs across countries with different income status.

**Methods:**

A cross-sectional web-based survey of 123 neurologists and headache specialists with at least 5 years of experience in headache management from 37 countries was carried out. They were classified into Low/Lower-Middle Income Countries (LIC/LMIC) and Upper-Middle Income Countries/High Income Countries (UMIC/HIC) groups in accordance with the World Bank income classifications.

**Results:**

Of 123 participants (median age 42 years; 50.4% males), 52.8% were from LICs/LMICs. Clinicians reported that CAMs were frequently used in both groups, and there was no significant difference in overall frequency of CAM use (*p* = 0.116). Clinicians in both groups perceived migraine as the most common headache type for which patients used CAM. However, significant differences in CAM modalities were observed. Acupuncture (79.3% vs. 47.7%, *p* < 0.001), massage (86.2% vs. 63.1%, *p* = 0.004), yoga (56.9% vs. 20.0%, *p* < 0.001), and supplements (93.1% vs. 66.2%, *p* < 0.001) were reported by the clinicians as significantly higher in UMICs/HICs, whereas spiritual healing was significantly higher in LICs/LMICs (70.8% vs. 46.6%, *p* = 0.006). Clinicians reported that patients in LICs/LMICs were significantly more likely to consult CAM prior to seeking medical care (89.2% vs. 67.2%, *p* = 0.003), and perceived cost as a major factor influencing CAM use (81.5% vs. 27.6%, *p* < 0.001).

**Conclusion:**

CAM appears to be commonly encountered in headache care across Africa and West Asia according to clinician reports. Although its use is shaped by cultural beliefs, accessibility, and perceived safety, concerns over unregulated products, herb-drug interactions, and delayed conventional medical treatments were also highlighted.

## Introduction

Strong evidence indicates a high epidemiological burden of headache disorders across African and West Asian countries (1–3). This burden is further amplified by the limited access to healthcare services, shortages of specialists, and high cost of conventional treatments in several low-income countries (4). These constraints often push patients toward seeking alternative pathways of care, including reliance on complementary practice and traditional remedies (5).

Substantial evidence indicates that complementary and alternative medicine (CAM) is commonly practiced in headache management (5). Its widespread use is mostly attributed to being a more accessible and affordable option, especially in settings where conventional medical services are limited or financially burdensome. Long-standing traditions and cultural beliefs further reinforce reliance on CAM. Additionally, CAM is frequently perceived as safer, more natural, and less likely to cause side effects (6). Dissatisfaction with conventional medical care also represents a major motivation for using CAM (7). A notable concern is the non-disclosure of many patients to their physicians about CAM use, posing challenges for providing them with optimal medical care (8).

In the last years, the prevalence of CAM use in African and West Asian countries has attracted considerable attention. Multiple epidemiological studies tried to explore this area. In sub-Saharan Africa, a substantial reliance on CAM has been reported either as a primary therapeutic approach or as an adjunct to conventional medical treatments (9). Likewise, in West Asia, a notable prevalence of CAM use was observed in both Qatar (10) and Saudi Arabia (11) with their use strongly influenced by religious and cultural beliefs.

Preliminary evidence denoted that the most commonly used CAM modalities in Africa and West Asian countries are herbal medicines, spiritual therapies, cupping, massage, relaxation techniques, mind–body methods, acupuncture, and dietary supplements (10, 12). Despite their widespread use, many of these modalities have not been formally evaluated. Such practices raise important concerns, including toxicity from unregulated products, herb–drug interactions, unqualified practitioners, and delay in seeking conventional medical care (9).

To address these gaps, it is crucial to explore the headache experts’ insights in Africa and West Asia regarding CAM use, identify the most frequently encountered modalities in clinical practice, and highlight the research challenges they recognize. Capturing clinician perspectives is fundamental to develop strategies for safe and evidence-based integration of CAM within headache care.

This study therefore aimed to assess the perspectives of clinicians from African and West Asian countries of varying income levels regarding the use of CAM in management of headache. This will help to provide insights into the prevalence, patterns, perceived effectiveness, safety considerations, and key research challenges associated with CAM use among headache patients.

## Methods

### Study design and participants

This cross-sectional study was carried out using an anonymous web-based survey. The survey link was sent via email to neurologists and headache specialists from several countries across Africa and West Asia to assess their insights and perspectives on the practice of CAM use in their respective countries. The survey was distributed by the study investigators who participated in study design and data collection. Invitations were sent through established professional networks, regional collaborations, national headache societies, and specialty contacts in the participating countries. Given the international nature of the study and the limited number of clinicians with expertise in headache medicine in several countries, purposive sampling rather than consecutive recruitment was employed to identify eligible neurologists and headache specialists with at least 5 years of clinical experience in headache management. The number of invitations varied across countries according to the availability of eligible clinicians and existing professional networks, rather than a fixed number of invitations assigned to each country. Recruitment was conducted between October 2025 and January 2026.

The countries list included Egypt, Ghana, Tunisia, Morocco, Nigeria, Tanzania, Republic of the Congo, Cameroon, Cote d’Ivoire, Benin, Kenya, Zambia, Zimbabwe, Namibia, Jordan, Mauritania, Djibouti, Burkina Faso, Chad, Central African Republic, Gambia, Malawi, Mozambique, Niger, Rwanda, Sudan, Uganda, Yemen, Turkey, Algeria, Azerbaijan, Libya, South Africa, Iran, Kuwait, Saudi Arabia, and United Arab Emirates. According to the World Bank (13), the included countries were categorized as low-income countries (LICs), lower-middle-income countries (LMICs), upper-middle/high-income countries (UMICs), and high-income countries (HICs) (Figure 1).

**Figure 1:**
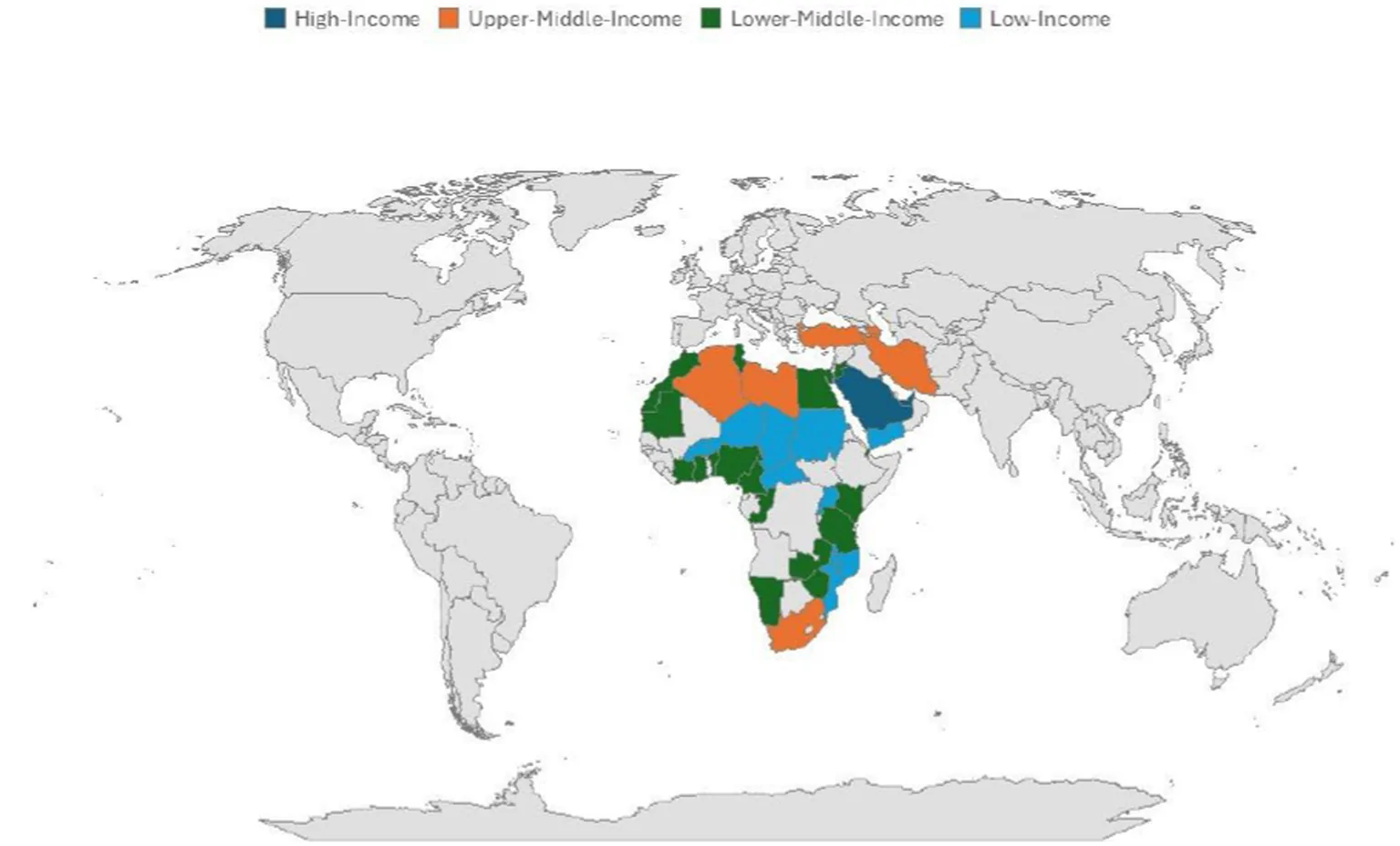
Geographic distribution of participating countries across Africa and West Asia, categorized according to World Bank income classification.

### Data collection

The draft questionnaire was created based on a comprehensive review of the relevant literature (14–17), and was tailored to address the study objectives. Prior to implementation, the draft questionnaire underwent pilot testing among 10 headache specialists to assess its clarity, comprehensibility, and applicability, and minor wording refinements were made accordingly.

The questionnaire was developed in English and administered exclusively in English to clinicians, therefore translation and cross-cultural adaptation procedures were not applicable. Likewise, because the instrument was developed for descriptive data collection rather than psychometric assessment, formal reliability testing (e.g., internal consistency or test–retest reliability) was not performed.

The final survey consisted of 17 closed-ended questions, divided into four: (i) Demographics and professional background of the participants (5 questions); (ii) type and frequency of CAM use in the headache practice (5 questions); (iii) their insights about patients’ beliefs and attitudes toward CAM use (3 questions); and (iv) their attitudes toward using CAM and their safety concerns, and revealing research gaps related to CAM use in headache management (4 questions).

CAM referred to the complementary and alternative medicine modalities listed in the questionnaire (cupping, herbal remedies, acupuncture, acupressure, massage and stretching therapy, relaxation exercises, yoga, hot or cold compresses, homeopathy, hydrotherapy, aromatherapy, dietary supplements and vitamins, as well as prayer and spiritual healing). Perceived effectiveness referred to the CAM modality that clinicians considered most effective in their clinical practice. Frequency of CAM use was assessed using a five-point ordinal scale (Never, Rarely, Sometimes, Often, and Always) based on clinicians’ observations of their patients.

Questions permitting multiple responses were analyzed using the total number of respondents as the denominator; therefore, percentages do not sum to 100%. Mandatory responses were required for all questions in the online survey platform; consequently, there were no missing data requiring special handling. The complete questionnaire, including all response options, was provided as Supplementary Material 1.

The survey requires approximately 8–10 min to complete. Two e-mail reminders were sent at 2-week intervals to the included participants.

### Study outcome

This study did not prespecify a single primary outcome. Instead, the survey was designed to capture clinicians’ perceptions across African and West Asian countries of varying income levels, regarding the frequency and types of CAM use in headache, perceived effectiveness, reasons for use, and timing of use, safety concerns, and research priorities related to CAM among patients with headache. Given the multiplicity of endpoints, all analyses were considered exploratory and hypothesis-generating.

### Sampling

Purposive sampling was used to identify and recruit eligible neurologists and headache specialists from countries across different World Bank income groups (13). Efforts were made to recruit clinicians from a broad range of countries and World Bank income groups to ensure geographic diversity. Of the 168 clinicians invited to participate, 123 from 37 countries completed the survey, corresponding to an overall response rate of 73.2%. The number of respondents per country was small and uneven, generally ranging from 2 to 7 clinicians, which provides broad geographic coverage but limits country-level representativeness. Country-specific numbers of clinicians invited and their response rates are presented in Supplementary Table 1.

### Statistical analysis

IBM SPSS (Statistical Package of Social Science) Version 25 was used to analyze the data. Kolmogorov–Smirnov test was used to test the normality of data. Categorical variables were expressed as numbers and percentages. Quantitative variables were expressed as median and inter quartile range (IQR). Chi-squared test was used for comparison between groups in categorical variables whereas Fisher’s exact test was applied instead of the chi-square test when expected cell counts were insufficient. Mann–Whitney U test was used for comparison between groups in quantitative variables. *p*-values were adjusted for multiple testing using Benjamini and Hochberg procedure. All tests were two-tailed.

## Results

### Demographics and professional background of the participants

This cross-sectional study was conducted on 123 clinicians from 37 African and West Asian countries. The median age was 42 (37–50) years. The sex distribution was nearly equal, with 62 males (50.4%) and 61 females (49.6%). Regarding country income classification, 65 participants (52.8%) were from LICs/LMICs, while 58 participants (47.2%) were from HICs/UMICs. In terms of workplace setting, nearly half of the participants were employed in university hospitals (47.2%), followed by general hospitals (25.2%). Smaller proportions worked in private hospitals (13.8%), private clinics (8.9%), and insurance hospitals (4.9%). The median years of experience in headache practice was 10 (7–20) years (Table 1).

**Table 1:** Demographics and professional background of the included participants.

Variables		Participants (*n* = 123)
Age [median (IQR)]		42 (37–50)
Sex [*n* (%)]	Males	62 (50.4%)
	Females	61 (49.6%)
Country according to income [*n* (%)]	LICs/LMICs^1^	65 (52.8%)
	HICs/UMICs^2^	58 (47.2%)
Workplace [*n* (%)]	General hospital	31 (25.2%)
	Insurance hospital	6 (4.9%)
	University hospital	58 (47.2%)
	Private hospital	17 (13.8%)
	Private clinic	11 (8.9%)
Years of experience in headache [median (IQR)]		10 (7–20)

HICs, high-income countries; LICs, low-income countries; LMICs, lower-middle-income countries; UMICs, upper-middle-income countries.

1 Egypt, Ghana, Tunisia, Morocco, Nigeria, Tanzania, Republic of the Congo, Cameroon, Cote d’Ivoire, Benin, Kenya, Zambia, Zimbabwe, Namibia, Jordan, Mauritania, Djibouti, Burkina Faso, Chad, Central African Republic, Gambia, Malawi, Mozambique, Niger, Rwanda, Sudan, Uganda, Yemen.

2 Turkey, Algeria, Azerbaijan, Libya, South Africa, Iran, Kuwait, Saudi Arabia, United Arab Emirates.

### Types, frequency, and indication of CAM use among headache patients

There were notable differences between LICs/LMICs and HICs/UMICs regarding the types of CAM used for headache management based on clinicians’ observations. In the HICs/UMICs group, significantly higher use was reported for acupuncture (79.3% vs. 47.7%, *p*-value <0.001), massage and stretching therapy (86.2% vs. 63.1%, *p*-value = 0.004), yoga (56.9% vs. 20.0%, *p*-value <0.001), aromatherapy (39.7% vs. 18.5%, *p*-value = 0.009), and dietary supplements/vitamins (93.1% vs. 66.2%, *p*-value <0.001). In contrast, prayer/spiritual healing was significantly more common in LICs/LMICs (70.8% vs. 46.6%, *p*-value = 0.006). No significant differences were observed for cupping, relaxation exercises, herbal remedies, acupressure, hot/cold compresses, homeopathy, or hydrotherapy (Table 2).

**Table 2:** Types, frequency, and indication of CAM use among headache patients.

Variables		LICs/LMICs group (*n* = 65)	HICs/UMICs group (*n* = 58)	*p*-value	Odds ratio	95% CI	
						Lower	Lower
The commonly used CAM in headache	Cupping (Hijama)	30 (46.2%)	39 (67.2%)	0.019	2.395	1.150	4.988
	Herbal remedies	54 (83.1%)	46 (79.3%)	0.593	0.781	0.315	1.936
	Acupuncture	31 (47.7%)	46 (79.3%)	<0.001*	4.204	1.888	9.360
	Acupressure	4 (6.2%)	6 (10.3%)	0.514	1.760	0.471	6.575
	Massage and stretching therapy	41 (63.1%)	50 (86.2%)	0.004*	3.659	1.487	9.002
	Relaxation exercise	35 (53.8%)	43 (74.1%)	0.020	2.457	1.145	5.274
	Yoga	13 (20.0%)	33 (56.9%)	<0.001*	5.280	2.373	11.747
	Hot or cold compresses	46 (70.8%)	43 (74.1%)	0.677	1.184	0.535	2.621
	Homeopathy	20 (30.8%)	23 (39.7%)	0.302	1.479	0.702	3.113
	Hydrotherapy	10 (15.4%)	15 (25.9%)	0.149	1.919	0.785	4.691
	Aromatherapy	12 (18.5%)	23 (39.7%)	0.009*	2.902	1.281	6.578
	Dietary supplements/vitamins	43 (66.2%)	54 (93.1%)	<0.001*	6.907	2.213	21.557
	Prayer/spiritual healing	46 (70.8%)	27 (46.6%)	0.006*	0.360	0.171	0.756
The most effective CAM for treatment of headache	Cupping (Hijama)	32 (49.2%)	28 (48.3%)	0.916	0.963	0.474	1.954
	Herbal remedies	50 (76.9%)	33 (56.9%)	0.018	0.396	0.182	0.861
	Acupuncture	38 (58.5%)	43 (74.1%)	0.067	2.037	0.946	4.387
	Aquapressure	15 (23.1%)	7 (12.1%)	0.112	0.458	0.172	1.217
	Massage and stretching therapy	33 (50.8%)	37 (63.8%)	0.145	1.709	0.829	3.522
	Relaxation exercise	37 (56.9%)	42 (72.4%)	0.074	1.986	0.932	4.234
	Yoga	28 (43.1%)	38 (65.5%)	0.013	2.511	1.209	5.215
	Hot or cold compresses	29 (44.6%)	31 (53.4%)	0.328	1.425	0.700	2.902
	Homeopathy	26 (40.0%)	17 (29.3%)	0.215	0.622	0.293	1.320
	Hydrotherapy	18 (27.7%)	19 (32.8%)	0.541	1.272	0.588	2.753
	Aromatherapy	19 (29.2%)	25 (43.1%)	0.109	1.834	0.870	3.865
	Dietary supplements/vitamins	46 (70.8%)	44 (75.9%)	0.525	1.298	0.581	2.902
	Prayer/spiritual healing	28 (43.1%)	19 (32.8%)	0.240	0.644	0.308	1.344
Frequency of using CAM in headache	Never	2 (3.1%)	3 (5.2%)	0.109	Ref	—	—
	Rarely	6 (9.2%)	15 (25.9%)		1.667	0.220	12.617
	Sometimes	32 (49.2%)	26 (44.8%)		0.542	0.084	3.488
	Often	23 (35.4%)	13 (22.4%)		0.377	0.056	2.555
	Always	2 (3.1%)	1 (1.7%)		0.333	0.017	6.654
Population categories frequently using CAM	Women	52 (80.0%)	54 (93.1%)	0.036	3.375	1.033	11.023
	Rural populations	59 (90.8%)	45 (77.6%)	0.043	0.352	0.124	0.998
	Older patients	47 (72.3%)	33 (56.9%)	0.074	0.506	0.238	1.072
	Lower socioeconomic groups	60 (92.3%)	45 (77.6%)	0.021	0.288	0.096	0.868
	Patients with low educational level	56 (86.2%)	41 (70.7%)	0.036	0.388	0.157	0.956
Headache type most often treated with CAM	Migraine	22 (33.8%)	33 (56.9%)	0.077	Ref	—	—
	Tension-type headache	24 (36.9%)	14 (24.1%)		0.389	0.166	0.911
	Chronic daily headache	15 (23.1%)	9 (15.5%)		0.400	0.149	1.073
	MOH	2 (3.1%)	2 (3.4%)		0.667	0.087	5.091
	Cluster headache	2 (3.1%)	0		0.000	0.000	

CAM, complementary and alternative medicine; HICs, high-income countries; LICs, low-income countries; LMICs, lower-middle-income countries; MOH, medication-overuse headache; UMICs, upper-middle-income countries.

*p*-values were adjusted for multiple testing using Benjamini and Hochberg procedure.

*p-value ≤0.009 is considered significant.

Bold values mean that they are statistically significant.

Regarding perceived effectiveness, none of the CAM modalities showed statistically significant differences between LICs/LMICs and HICs/UMICs groups (Table 2).

No perceived differences in the frequency of CAM use between the two groups according to clinician reports (*p*-value = 0.109). However, “sometimes” and “often” were the most commonly reported frequencies in both groups.

Regarding population groups who were more frequently use CAM, most clinicians in both groups perceived women (93.1% vs. 80.0%, *p*-value = 0.036), rural populations (90.8% vs. 77.6%, *p*-value = 0.043), lower socioeconomic groups (92.3% vs. 77.6%, *p*-value = 0.021), patients with lower educational levels (86.2% vs. 70.7%, *p*-value = 0.036), and older patients (72.3% vs. 56.9%, *p*-value = 0.074) as more likely to use CAM, with no significant differences observed between LICs/LMICs and HICs/UMICs groups (Table 2).

As for headache type, clinicians reported that migraine was the most commonly reported type treated with CAM in both groups, followed by tension-type headache and chronic daily headache, with no statistically significant differences between the two groups (*p*-value = 0.077) (Table 2).

Further descriptive analyses of the types, frequency, and indications of CAM use among headache patients across all four World Bank income categories are presented in Supplementary Table 2.

### Patient beliefs and behaviors regarding CAM use

Based on clinicians’ observations, the reasons for CAM use were generally comparable between LICs/LMICs and HICs/UMICs, with cultural or religious beliefs (90.8% vs. 91.4%) and advice from others (95.4% vs. 86.2%) being highly reported in both groups. However, high cost of medications was significantly more frequently reported as a reason in LICs/LMICs compared to HICs/UMICs (81.5% vs. 27.6%, *p*-value <0.001). Other factors, including dissatisfaction with conventional treatments, fewer side effects, and accessibility, did not differ significantly between the two groups (Table 3).

**Table 3:** Clinician-reported patient beliefs and behaviors regarding CAM use.

Variables		LICs/LMICs group (*n* = 65)	HICs/UMICs group (*n* = 58)	*p*-value	Odds ratio	95% CI	
						Lower	Upper
Main reasons for CAM use among headache patients	Dissatisfaction with conventional treatments	48 (73.8%)	50 (86.2%)	0.089	2.214	0.874	5.605
	High cost of medications	53 (81.5%)	16 (27.6%)	<0.001*	0.086	0.037	0.202
	Cultural or religious beliefs	59 (90.8%)	53 (91.4%)	0.906	1.078	0.311	3.738
	Fewer side effects	30 (46.2%)	30 (51.7%)	0.537	1.250	0.615	2.541
	Accessibility	52 (80.0%)	38 (65.5%)	0.070	0.475	0.210	1.072
	Advice from others	62 (95.4%)	50 (86.2%)	0.075	0.302	0.076	1.200
The stage at which patients usually seek CAM	Before consulting a medical professional	58 (89.2%)	39 (67.2%)	0.003*	0.248	0.095	0.645
	After diagnosis but alongside conventional treatment	42 (64.6%)	36 (62.1%)	0.770	0.896	0.430	1.868
	After failure or side effects of medical therapy	49 (75.4%)	43 (74.1%)	0.874	0.936	0.414	2.114
	As a preventive measure	28 (43.1%)	32 (55.2%)	0.180	1.626	0.797	3.319
Patient disclosure of CAM use to physicians	Never	2 (3.1%)	1 (1.7%)	0.687	Ref	—	—
	Rarely	10 (15.4%)	13 (22.4%)		2.600	0.205	32.904
	Sometimes	35 (53.8%)	28 (48.3%)		1.600	0.138	18.566
	Often	16 (24.6%)	12 (20.7%)		1.500	0.121	18.540
	Always	2 (3.1%)	4 (6.9%)		4.000	0.211	75.659

CAM, complementary and alternative medicine; HICs, high-income countries; LICs, Low-income countries; LMICs, lower-middle-income countries; UMICs, upper-middle-income countries.

*p*-values were adjusted for multiple testing using Benjamini and Hochberg procedure.

^*^p-value ≤0.003 is considered significant.

Bold values mean that they are statistically significant.

Regarding the timing of CAM use, patients in LICs/LMICs were significantly more likely to seek CAM before consulting a medical professional compared to those in HICs/UMICs according to clinician reports (89.2% vs. 67.2%, *p*-value = 0.003). No significant differences were observed for CAM use after diagnosis alongside conventional treatment, after failure or side effects of medical therapy, or as a preventive measure (Table 3).

In terms of disclosure of CAM use to physicians, there were no significant differences between the two groups (*p*-value = 0.687). In both groups, clinicians reported that the majority of patients reported “sometimes” disclosing CAM use (53.8% in LICs/LMICs vs. 48.3% in HICs/UMICs), followed by “often” (24.6% in LICs/LMICs vs. 20.7% in HICs/UMICs), while only a small proportion reported always or never disclosing such use (Table 3).

Further descriptive analyses of patient beliefs and behaviors regarding CAM use across all four World Bank income categories are presented in Supplementary Table 3.

### Safety concerns, research challenges, and priority areas related to CAM use in headache management

The reported safety concerns related to CAM use were comparable between LICs/LMICs and HICs/UMICs according to clinician reports, with no statistically significant differences. The most frequently identified concerns in both groups included delay in seeking appropriate medical care (87.7% vs. 82.8%), lack of scientific evidence (86.2% vs. 82.8%), herb-drug interactions (84.6% vs. 79.3%), unqualified practitioners (84.6% vs. 87.9%), and contamination or toxicity of CAM products (80.0% vs. 84.5%) (Table 4).

**Table 4:** Safety concerns, research challenges, and priority areas related to CAM use in headache management.

Variables		LICs/LMICs group (*n* = 65)	HICs/UMICs group (*n* = 58)	*p*-value	Odds ratio	95% CI	
						Lower	Upper
Main safety concerns regarding CAM use in headache patients	Herb–drug interactions	55 (84.6%)	46 (79.3%)	0.443	0.697	0.276	1.760
	Contamination or toxicity from CAM products	52 (80.0%)	49 (84.5%)	0.517	1.361	0.534	3.468
	Unqualified practitioners	55 (84.6%)	51 (87.9%)	0.595	1.325	0.469	3.741
	Delay in seeking appropriate medical care	57 (87.7%)	48 (82.8%)	0.440	0.674	0.246	1.842
	Lack of scientific evidence	56 (86.2%)	48 (82.8%)	0.603	0.771	0.290	2.055
The main challenges in studying CAM use	Lack of research funding	63 (96.9%)	44 (75.9%)	0.001*	0.100	0.022	0.461
	Lack of standardized CAM practices	60 (92.3%)	53 (91.4%)	0.851	0.883	0.242	3.220
	Poor documentation of traditional practices	56 (86.2%)	53 (91.4%)	0.362	1.704	0.536	5.412
	Patient underreporting of CAM use	53 (81.5%)	31 (53.4%)	0.001*	0.260	0.115	0.585
	Limited collaboration between traditional practitioners and clinicians	60 (92.3%)	53 (91.4%)	0.851	0.883	0.242	3.220
Recommended research priorities regarding CAM use in headache	Epidemiological studies to assess prevalence and patterns of CAM use	64 (98.5%)	45 (77.6%)	<0.001*	0.054	0.007	0.428
	Clinical trials evaluating safety and efficacy of CAM	55 (84.6%)	48 (82.8%)	0.781	0.873	0.335	2.275
	Education programs to train healthcare providers about CAM	54 (83.1%)	51 (87.9%)	0.447	1.484	0.534	4.124
	Implementation studies integrating CAM and conventional treatment	51 (78.5%)	51 (87.9%)	0.164	2.000	0.746	5.365
CAM modalities prioritized for future scientific evaluation in headache	Cupping (Hijama)	24 (36.9%)	19 (32.8%)	0.629	0.832	0.395	1.752
	Herbal remedies	24 (36.9%)	13 (22.4%)	0.080	0.494	0.223	1.095
	Acupuncture	22 (33.8%)	34 (58.6%)	0.006*	2.769	1.331	5.762
	Acupressure	4 (6.2%)	3 (5.2%)	1.000	0.832	0.178	3.883
	Massage and stretching therapy	33 (50.8%)	37 (63.8%)	0.145	1.709	0.829	3.522
	Relaxation exercise	41 (63.1%)	49 (84.5%)	0.007*	3.187	1.334	7.616
	Yoga	15 (23.1%)	30 (51.7%)	0.001*	3.571	1.648	7.740
	Hot or cold compresses	26 (40.0%)	25 (43.1%)	0.727	1.136	0.554	2.331
	Homeopathy	4 (6.2%)	3 (5.2%)	1.000	0.832	0.178	3.883
	Hydrotherapy	7 (10.8%)	7 (12.1%)	1.000	1.137	0.374	3.462
	Aromatherapy	7 (10.8%)	9 (15.5%)	0.593	1.522	0.528	4.386
	Dietary supplements/vitamins	31 (47.7%)	37 (63.8%)	0.073	1.932	0.937	3.984
	Prayer/spiritual healing	27 (41.5%)	12 (20.7%)	0.013*	0.367	0.164	0.821

CAM, complementary and alternative medicine; HICs, high-income countries; LICs, low-income countries; LMICs, lower-middle-income countries; UMICs, upper-middle-income countries.

*p*-values were adjusted for multiple testing using Benjamini and Hochberg procedure.

^*^p-value ≤0.013 is considered significant.

Bold values mean that they are statistically significant.

Significant differences emerged regarding challenges in studying CAM. Lack of research funding (96.9% vs. 75.9%, *p*-value = 0.001) and patient underreporting of CAM use (81.5% vs. 53.4%, *p*-value = 0.001) was more frequently reported by clinicians from LICs/LMICs compared to HICs/UMICs. Other challenges, including lack of standardized CAM practices, poor documentation of traditional practices, and limited collaboration between traditional practitioners and clinicians, were similarly reported in both groups (Table 4).

In terms of recommended research priorities, clinicians from LICs/LMICs more frequently emphasized the need for epidemiological studies to assess prevalence and patterns of CAM use (98.5% vs. 77.6%, *p*-value < 0.001). No significant differences were observed for clinical trials evaluating safety and efficacy, education programs for healthcare providers, or implementation studies integrating CAM with conventional treatment (Table 4).

Regarding CAM therapies that deserve priority for scientific evaluation, acupuncture (58.6% vs. 33.8%, *p*-value = 0.006), relaxation exercises (84.5% vs. 63.1%, *p*-value = 0.007), and yoga (51.7% vs. 23.1%, *p*-value = 0.001) were significantly more emphasized in HICs/UMICs. In contrast, prayer/spiritual healing was more frequently prioritized in LICs/LMICs (41.5% vs. 20.7%, *p*-value = 0.013). No significant differences were observed for other CAM modalities (Table 4).

Further descriptive analyses of safety concerns, research challenges, and priority areas related to CAM use in headache management across all four World Bank income categories are presented in Supplementary Table 4.

## Discussion

In the present study, clinicians consistently reported CAM to be commonly used among headache patients across Africa and West Asia. According to clinicians’ perceptions, CAM use appears to be influenced by a complex interplay of cultural beliefs, healthcare accessibility, and economic factors that may encourage patients to seek alternative forms of therapy.

Surprisingly, our findings revealed no significant difference in the rate of CAM utilization between headache patients from higher and lower income countries (*p* = 0.116), thus confirming that CAM utilization is a universal practice likely driven by universal factors, including cultural beliefs, perceived efficacy, and healthcare needs.

However, we found that patients in HICs/UMICs used modalities such as acupuncture, massage and stretching, yoga, aromatherapy, and supplements, while prayer and spiritual healing were used by patients in LICs/LMICs. This finding suggests that income level may influence the choice of CAM modality. However, differences observed between income groups likely reflect a broader interplay of healthcare systems, clinician background, and workplace setting. Cultural practices, and religion rather than income alone.

Moreover, the emphasis on prayer and spiritual healing in LICs/LMICs could be a reflection of the influence of cultural and religious practices on health behaviors in these regions. This could be supported by the observations of studies that have sought to understand health behaviors in regions such as Sub-Saharan Africa and parts of the Middle East, where spiritual and traditional healing practices are major aspects of the health behaviors in the region (18, 19). Such practices are considered accessible and affordable in the region, making them the most sought-after health interventions in the region (9).

The motivations for CAM practice differed between the two income groups. In LICs/LMICs, the high cost of conventional medical treatment was found to be an important factor in CAM practice, which again correlates with previous findings emphasizing financial barriers and inequity in headache care in these regions (9). On the other hand, cultural beliefs, advice from family and social circles, and safety were consistently reported in both income groups. These findings again correlate with previous research findings, where it has been emphasized that CAM practice is often influenced by social networks and is viewed as “natural and safe with fewer side effects” (20).

Another interesting finding is that most patients in LICs/LMICs tend to access CAM services prior to consulting professional medical facilities. It is noteworthy that this trend is concerning, as it can lead to worsening of the headache condition and can even cause chronic headache disorders (21). Similar trends have been noticed in Sub-Saharan Africa, wherein traditional medicine and CAM are often the first line of treatment and can act as a barrier to evidence-based practices (22). In this context, it is essential that to improve access to healthcare facilities and to enhance public awareness regarding the need for early medical consultation for better headache control.

In this study, migraine was the most common type of headache treated with CAM in both groups. Tension-type and chronic daily headache were next, and there was no significant difference between the two income groups. This is in line with previous research that shows migraine sufferers are more likely to seek CAM because the condition is long-lasting and debilitating, alongside dissatisfaction or inadequacies with conventional treatments (21). The similar pattern across different countries suggests that disease-related factors, rather than socioeconomic differences, are the main drivers of CAM use. Furthermore, the presence of chronic headache types reinforces the inclination of patients with persistent symptoms to seek multimodal management strategies, including CAM (23).

The majority of participants indicated that patients only disclose the use of CAM at times, with no significant differences in findings between the different income groups. This finding corroborates earlier research indicating that nondisclosure regarding the use of CAM is quite common, with patients citing fear of being disapproved of or the belief that physicians are not knowledgeable regarding these practices. Such nondisclosure poses serious risk, especially with regard to herb–drug interaction or adverse effects (8, 24). This situation calls for the development of effective communication skills by clinicians to encourage patients to discuss CAM use.

The current research has many notable strengths. First, the study included a wide range of countries across Africa and West Asia. Second, the sample was representative of both LICs/LMICs and HICs/UMICs, offering novel comparative insights into how economic context shapes CAM use. Third, the study goes beyond simple prevalence of CAM by providing comprehensive, multidimensional evaluation that encompasses patterns of its use, perceived effectiveness, patient behaviors, safety considerations, and research priorities.

There are still several limitations with this study. First, the findings are based on clinicians’ self-reported perceptions rather than direct patient-level data, which may be subject to recall, observer, and social desirability bias, potentially resulting in under- or over-reporting of CAM use. Second, because patient data were not collected, the reported patterns of CAM use could not be independently verified. Third, comparisons across country income groups should not be interpreted as patient-level or causal inferences, and the potential for ecological inference should be considered when interpreting the findings. Fourth, the analyses were not weighted according to the number of clinicians or population size of each country; therefore, countries contributing larger numbers of respondents may have had a greater influence on the overall findings. Fifth, the number of respondents per country was small and uneven, which limited representativeness and precluded cluster-adjusted analyses. As a result, within-country correlation could not be formally accounted for. Therefore, our findings should be interpreted as descriptive summaries of clinician perceptions rather than inferential comparisons across income groups. Sixth, classification according to World Bank income did not not account for important differences in culture, religion, healthcare systems, clinician characteristics, or other contextual factors. Consequently, the observed differences should not be attributed to income level alone and may be influenced by residual confounding and within-group heterogeneity. Lastly, the study did not assess the clinical outcomes or efficacy of CAM modalities.

## Conclusion

According to clinicians’ reports, CAM plays an important role in headache management across Africa and West Asia. Clinicians perceived that its use is shaped by cultural, economic, and healthcare-access factors rather than income level alone. They also highlighted important concerns related to safety, delayed medical consultation, and limited scientific evidence supporting many CAM modalities. The observed differences between the income groups in the present study are best regarded as hypothesis-generating and should not be attributed directly to income level or healthcare resources.

## Data Availability

The raw data supporting the conclusions of this article will be made available by the authors, without undue reservation.
