# Supplementary material for: Clinicians’ perspectives on complementary and alternative medicine use in headache care across Africa and West Asia: a cross-sectional survey
Source: Front Neurol. 2026 Oct 13;17:1849417. doi: 10.3389/fneur.2026.1849417 (PMC13710024; doi:10.3389/fneur.2026.1849417)
Supplement: Supplementary file 1 [file Supplementary_file_1.docx]

**Complementary and Alternative Medicine (CAM) in Headache Care Across Africa and West Asia: A Cross-sectional Survey of Clinician Perspectives**

1. **Age**
2. **Sex**
3. **Country**
4. **Workplace**

- General hospital
- Insurance hospital
- University hospital
- Private hospital
- Private clinic
- Primary healthcare center

5. **Years of experience in Headache**
6. **What are the commonly used CAM in your country?**

- Cupping (Hijama)
- Herbal remedies
- Acupuncture
- Acupressure
- Massage and stretching therapy
- Relaxation exercise
- Yoga
- Hot or cold compresses
- Homeopathy
- Hydrotherapy
- Aromatherapy
- Dietary supplements/vitamins
- Prayer/spiritual healing
- Other CAM (please specify)

7. **What is the most effective CAM treatment for headache you have observed in your practice?**

- Cupping (Hijama)
- Herbal remedies
- Acupuncture
- Acupressure
- Massage and stretching therapy
- Relaxation exercise
- Yoga
- Hot or cold compresses
- Homeopathy
- Hydrotherapy
- Aromatherapy
- Dietary supplements/vitamins
- Prayer/spiritual healing
- Other CAM (please specify)

8. **How often do your patients use CAM?**

- Never
- Rarely
- Sometimes
- Often
- Always

9. **Do you think CAM use is more common among the following groups?**

- Women
- Rural populations
- Older patients
- Lower socioeconomic groups
- Patients with low educational level
- Other (please specify)

10. **What is the most common headache type most often treated with CAM in your country?**

- Migraine
- Tension‑type headache
- Chronic daily headache
- Medication‑overuse headache
- Cluster headache
- Other (please specify)

11. **In your opinion, what are the main reasons for which patients with headache choose CAM?**

- Dissatisfaction with conventional treatments
- High cost of medications
- Cultural or religious beliefs
- Fewer side effects
- Accessibility
- Advice from others
- Other (please specify)

12. **At what stage do patients usually seek CAM?**

- Before consulting a medical professional
- After diagnosis but alongside conventional treatment
- After failure or side effects of medical therapy
- As a preventive measure

13. **Do patients typically disclose CAM use to you?**

- Never
- Rarely
- Sometimes
- Often
- Always

14. **What are your main safety concerns regarding CAM use in headache patients?**

- Herb-drug interactions
- Contamination or toxicity from some products
- Unqualified practitioners
- Delay in seeking appropriate medical care
- Lack of scientific evidence
- Other (please specify)

15. **What are the main challenges in studying CAM use among headache patients in your country?**

- Lack of research funding
- Lack of standardized CAM practices
- Poor documentation of traditional practices
- Patient underreporting of CAM use
- Limited collaboration between traditional practitioners and clinicians
- Other (please specify)

16. **What research priorities would you recommend regarding CAM and headache in your country?**

- Epidemiological studies to assess prevalence and patterns of CAM use
- Clinical trials evaluating safety and efficacy of CAM
- Education programs to train healthcare providers about CAM
- Implementation studies integrating CAM and conventional care
- Other (please specify)

17. **Which CAM therapies deserve priority for scientific evaluation in your country?**

- Cupping (Hijama)
- Herbal remedies
- Acupuncture
- Acupressure
- Massage and stretching therapy
- Relaxation exercise
- Yoga
- Hot or cold compresses
- Homeopathy
- Hydrotherapy
- Aromatherapy
- Dietary supplements/vitamins
- Prayer/spiritual healing
- Other (please specify)
