# Supplementary material for: Clinicians’ perspectives on complementary and alternative medicine use in headache care across Africa and West Asia: a cross-sectional survey
Source: Front Neurol. 2026 Oct 13;17:1849417. doi: 10.3389/fneur.2026.1849417 (PMC13710024; doi:10.3389/fneur.2026.1849417)
Supplement: Supplementary file 2 [file Table_1.docx]

**Supplementary Table (1):** Country‑specific numbers of clinicians invited and their response rates

| Countries | The number of clinicians who were invited (n=168) | The number of clinicians who responded  (n=123) | Response rate |
| --- | --- | --- | --- |
| High-income countries | | | |
| Kuwait | 5 | 5 | 100% |
| Saudi Arabia | 7 | 7 | 100% |
| United Arab Emirates | 6 | 6 | 100% |
| Upper-middle-income countries | | | |
| Libya | 7 | 7 | 100% |
| South Africa | 6 | 6 | 100% |
| Algeria | 7 | 7 | 100% |
| Azerbaijan | 7 | 7 | 100% |
| Turkey | 7 | 7 | 100% |
| Iran | 6 | 6 | 100% |
| Lower-middle-income countries | | | |
| Egypt | 6 | 4 | 67% |
| Ghana | 4 | 3 | 75% |
| Tunisia | 5 | 4 | 80% |
| Morocco | 4 | 3 | 75% |
| Nigeria | 4 | 3 | 75% |
| Tanzania | 4 | 2 | 50% |
| Republic of the Congo | 4 | 2 | 50% |
| Cameroon | 4 | 2 | 50% |
| Cote d'Ivoire | 4 | 2 | 50% |
| Benin | 4 | 2 | 50% |
| Kenya | 5 | 2 | 40% |
| Zambia | 4 | 2 | 50% |
| Zimbabwe | 4 | 2 | 50% |
| Namibia | 4 | 2 | 50% |
| Jordan | 4 | 2 | 50% |
| Mauritania | 4 | 2 | 50% |
| Low-income countries | | | |
| Burkina Faso | 4 | 2 | 50% |
| Djibouti | 3 | 2 | 67% |
| Chad | 3 | 2 | 67% |
| Central African Republic | 4 | 2 | 50% |
| Gambia | 3 | 2 | 67% |
| Malawi | 3 | 2 | 67% |
| Mozambique | 3 | 2 | 67% |
| Niger | 4 | 2 | 50% |
| Rwanda | 3 | 2 | 67% |
| Sudan | 4 | 3 | 75% |
| Uganda | 4 | 2 | 50% |
| Yemen | 4 | 3 | 75% |
