# Supplementary material for: Clinicians’ perspectives on complementary and alternative medicine use in headache care across Africa and West Asia: a cross-sectional survey
Source: Front Neurol. 2026 Oct 13;17:1849417. doi: 10.3389/fneur.2026.1849417 (PMC13710024; doi:10.3389/fneur.2026.1849417)
Supplement: Supplementary file 3 [file Table_2.docx]

**Supplementary Table (2):** Types, frequency, and indication of CAM use among headache patients in the four income groups

|  | | HICs  group  (n= 18) | UMICs group  (n= 40) | LMICs group  (n= 39) | LICs  group  (n= 26) |
| --- | --- | --- | --- | --- | --- |
| The commonly used CAM in headache | Cupping (Hijama) | 12 (66.7%) | 27 (67.5%) | 16 (41.0%) | 14 (53.8%) |
|  | Herbal remedies | 13 (72.2%) | 33 (82.5%) | 34 (87.2%) | 20 (76.9%) |
|  | Acupuncture | 16 (88.9%) | 30 (75.0%) | 18 (46.2%) | 13 (50.0%) |
|  | Acupressure | 2 (11.1%) | 4 (10.0%) | 3 (7.7%) | 1 (3.8%) |
|  | Massage and stretching therapy | 15 (83.3%) | 35 (87.5%) | 26 (66.7%) | 15 (57.7%) |
|  | Relaxation exercise | 16 (88.9%) | 27 (67.5%) | 24 (61.5%) | 11 (42.3%) |
|  | Yoga | 12 (66.7%) | 21 (52.5%) | 9 (23.1%) | 4 (15.4%) |
|  | Hot or cold compresses | 12 (66.7%) | 31 (77.5%) | 26 (66.7%) | 20 (76.9%) |
|  | Homeopathy | 8 (44.4%) | 15 (37.5%) | 18 (46.2%) | 2 (7.7%) |
|  | Hydrotherapy | 2 (11.1%) | 13 (32.5%) | 6 (15.4%) | 4 (15.4%) |
|  | Aromatherapy | 6 (33.3%) | 17 (42.5%) | 8 (20.5%) | 4 (15.4%) |
|  | Dietary supplements/vitamins | 17 (94.4%) | 37 (92.5%) | 30 (76.9%) | 13 (50.0%) |
|  | Prayer/spiritual healing | 6 (33.3%) | 21 (52.5%) | 28 (71.8%) | 18 (69.2%) |
| The most effective CAM for treatment of headache | Cupping (Hijama) | 7 (38.9%) | 21 (52.5%) | 16 (41.0%) | 16 (61.5%) |
|  | Herbal remedies | 7 (38.9%) | 26 (65.0%) | 35 (89.7%) | 15 (57.7%) |
|  | Acupuncture | 13 (72.2%) | 30 (75.0%) | 25 (64.1%) | 13 (50.0%) |
|  | Aquapressure | 4 (22.2%) | 3 (7.5%) | 10 (25.6%) | 5 (19.2%) |
|  | Massage and stretching therapy | 14 (77.8%) | 23 (57.5%) | 23 (59.0%) | 10 (38.5%) |
|  | Relaxation exercise | 14 (77.8%) | 28 (70.0%) | 26 (66.7%) | 11 (42.3%) |
|  | Yoga | 12 (66.7%) | 26 (65.0%) | 22 (56.4%) | 6 (23.1%) |
|  | Hot or cold compresses | 9 (50.0%) | 22 (55.0%) | 20 (51.3%) | 9 (34.6%) |
|  | Homeopathy | 4 (22.2%) | 13 (32.5%) | 19 (48.7%) | 7 (26.9%) |
|  | Hydrotherapy | 5 (27.8%) | 14 (35.0%) | 10 (25.6%) | 8 (30.8%) |
|  | Aromatherapy | 10 (55.6%) | 15 (37.5%) | 14 (35.9%) | 5 (19.2%) |
|  | Dietary supplements/vitamins | 15 (83.3%) | 29 (72.5%) | 31 (79.5%) | 15 (57.7%) |
|  | Prayer/spiritual healing | 4 (22.2%) | 15 (37.5%) | 19 (48.7%) | 9 (34.6%) |
| Frequency of using CAM in headache | Never | 1 (5.6%) | 2 (5.0%) | 1 (2.6%) | 1 (3.8%) |
|  | Rarely | 9 (50.0%) | 6 (15.0%) | 3 (7.7%) | 3 (11.5%) |
|  | Sometimes | 5 (27.8%) | 21 (52.5%) | 19 (48.7%) | 13 (50.0%) |
|  | Often | 3 (16.7%) | 10 (25.0%) | 15 (38.5%) | 8 (30.8%) |
|  | Always | 0 | 1 (2.5%) | 1 (2.6%) | 1 (3.8%) |
| Population categories frequently using CAM | Women | 16 (88.9%) | 38 (95.0%) | 29 (74.4%) | 23 (88.5%) |
|  | Rural populations | 13 (72.2%) | 32 (80.0%) | 35 (89.7%) | 24 (92.3%) |
|  | Older patients | 11 (61.1%) | 22 (55.0%) | 28 (71.8%) | 19 (73.1%) |
|  | Lower socioeconomic groups | 12 (66.7%) | 33 (82.5%) | 36 (92.3%) | 24 (92.3%) |
|  | Patients with low educational level | 12 (66.7%) | 29 (72.5%) | 31 (79.5%) | 25 (96.2%) |
| Headache type most often treated with CAM | Migraine | 14 (77.8%) | 19 (47.5%) | 14 (35.9%) | 8 (30.8%) |
|  | Tension-type headache | 2 (11.1%) | 12 (30.0%) | 13 (33.3%) | 11 (42.3%) |
|  | Chronic daily headache | 2 (11.1%) | 7 (17.5%) | 10 (25.6%) | 5 (19.2%) |
|  | MOH | 0 | 2 (5.0%) | 1 (2.6%) | 1 (3.8%) |
|  | Cluster headache | 0 | 0 | 1 (2.6%) | 1 (3.8%) |

CAM: Complementary and alternative medicine, HICs: High-income countries, LICs: Low-income countries, LMICs: Lower-middle-income countries, MOH: Medication-overuse headache, UMICs: Upper-middle-income countries
