# Supplementary material for: Clinicians’ perspectives on complementary and alternative medicine use in headache care across Africa and West Asia: a cross-sectional survey
Source: Front Neurol. 2026 Oct 13;17:1849417. doi: 10.3389/fneur.2026.1849417 (PMC13710024; doi:10.3389/fneur.2026.1849417)
Supplement: Supplementary file 4 [file Table_3.docx]

**Supplementary Table (3):** Clinician-reported patient beliefs and behaviors regarding CAM use in the four income groups

|  | | HICs  group  (n= 18) | UMICs group  (n= 40) | LMICs group  (n= 39) | LICs  group  (n= 26) |
| --- | --- | --- | --- | --- | --- |
| Main reasons for CAM use among headache patients | Dissatisfaction with conventional treatments | 16 (88.9%) | 34 (85.0%) | 32 (82.1%) | 16 (61.5%) |
|  | High cost of medications | 2 (11.1%) | 14 (35.0%) | 32 (82.1%) | 21 (80.8%) |
|  | Cultural or religious beliefs | 17 (94.4%) | 36 (90.0%) | 36 (92.3%) | 23 (88.5%) |
|  | Fewer side effects | 7 (38.9%) | 23 (57.5%) | 21 (53.8%) | 9 (34.6%) |
|  | Accessibility | 10 (55.6%) | 28 (70.0%) | 31 (79.5%) | 21 (80.8%) |
|  | Advice from others | 17 (94.4%) | 33 (82.5%) | 38 (97.4%) | 24 (92.3%) |
| The stage at which patients usually seek CAM | Before consulting a medical professional | 13 (72.2%) | 26 (65.0%) | 36 (92.3%) | 22 (84.6%) |
|  | After diagnosis but alongside conventional treatment | 16 (88.9%) | 20 (50.0%) | 24 (61.5%) | 18 (69.2%) |
|  | After failure or side effects of medical therapy | 12 (66.7%) | 31 (77.5%) | 31 (79.5%) | 18 (69.2%) |
|  | As a preventive measure | 8 (44.4%) | 24 (60.0%) | 18 (46.2%) | 10 (38.5%) |
| Patient disclosure of CAM use to physicians | Never | 0 | 1 (2.5%) | 0 | 2 (7.7%) |
|  | Rarely | 3 (16.7%) | 10 (25.0%) | 6 (15.4%) | 4 (15.4%) |
|  | Sometimes | 10 (55.6%) | 18 (45.0%) | 22 (56.4%) | 13 (50.0%) |
|  | Often | 3 (16.7%) | 9 (22.5%) | 9 (23.1%) | 7 (26.9%) |
|  | Always | 2 (11.1%) | 2 (5.0%) | 2 (5.1%) | 0 |

CAM: Complementary and alternative medicine, HICs: High-income countries, LICs: Low-income countries, LMICs: Lower-middle-income countries, UMICs: Upper-middle-income countries.
