# Supplementary material for: Clinicians’ perspectives on complementary and alternative medicine use in headache care across Africa and West Asia: a cross-sectional survey
Source: Front Neurol. 2026 Oct 13;17:1849417. doi: 10.3389/fneur.2026.1849417 (PMC13710024; doi:10.3389/fneur.2026.1849417)
Supplement: Supplementary file 5 [file Table_4.docx]

**Supplementary Table (4):** Safety concerns, research challenges, and priority areas related to CAM use in headache management in the four income groups

|  | | HICs  group  (n= 18) | UMICs group  (n= 40) | LMICs group  (n= 39) | LICs  group  (n= 26) |
| --- | --- | --- | --- | --- | --- |
| Main safety concerns regarding CAM use in headache patients | Herb–drug interactions | 14 (77.8%) | 32 (80.0%) | 33 (84.6%) | 22 (84.6%) |
|  | Contamination or toxicity from CAM products | 16 (88.9%) | 33 (82.5%) | 29 (74.4%) | 23 (88.5%) |
|  | Unqualified practitioners | 17 (94.4%) | 34 (85.0%) | 34 (87.2%) | 21 (80.8%) |
|  | Delay in seeking appropriate medical care | 18 (100%) | 30 (75.0%) | 33 (84.6%) | 24 (92.3%) |
|  | Lack of scientific evidence | 14 (77.8%) | 34 (85.0%) | 34 (87.2%) | 22 (84.6%) |
| The main challenges in studying CAM use | Lack of research funding | 11 (61.1%) | 33 (82.5%) | 39 (100.0%**)** | 24 (92.3%) |
|  | Lack of standardized CAM practices | 17 (94.4%) | 36 (90.0%) | 36 (92.3%) | 24 (92.3%) |
|  | Poor documentation of traditional practices | 17 (94.4%) | 36 (90.0%) | 32 (82.1%) | 24 (92.3%) |
|  | Patient underreporting of CAM use | 9 (50.0%) | 22 (55.0%) | 29 (74.4%) | 24 (92.3%) |
|  | Limited collaboration between traditional practitioners and clinicians | 16 (88.9%) | 37 (92.5%) | 37 (94.9%) | 23 (88.5%) |
| Recommended research priorities regarding CAM use in headache | Epidemiological studies to assess prevalence and patterns of CAM use | 14 (77.8%) | 31 (77.5%) | 39 (100.0%) | 25 (96.2%) |
|  | Clinical trials evaluating safety and efficacy of CAM | 15 (83.3%) | 33 (82.5%) | 34 (87.2%) | 21 (80.8%) |
|  | Education programs to train healthcare providers about CAM | 17 (94.4%) | 34 (85.0%) | 30 (76.9%) | 24 (92.3%) |
|  | Implementation studies integrating CAM and conventional treatment | 18 (100.0%) | 33 (82.5%) | 29 (74.4%) | 22 (84.6%) |
| CAM modalities prioritized for future scientific evaluation in headache | Cupping (Hijama) | 3 (16.7%) | 16 (40.0%) | 12 (30.8%) | 12 (46.2%) |
|  | Herbal remedies | 1 (5.6%) | 12 (30.0%) | 15 (38.5%) | 9 (34.6%) |
|  | Acupuncture | 10 (55.6%) | 24 (60.0%) | 10 (25.6%) | 12 (46.2%) |
|  | Acupressure | 1 (5.6%) | 2 (5.0%) | 3 (7.7%) | 1 (3.8%) |
|  | Massage and stretching therapy | 13 (72.2%) | 24 (60.0%) | 19 (48.7%) | 14 (53.8%) |
|  | Relaxation exercise | 15 (83.3%) | 34 (85.0%) | 26 (66.7%) | 15 (57.7%) |
|  | Yoga | 11 (61.1%) | 19 (47.5%) | 10 (25.6%) | 5 (19.2%) |
|  | Hot or cold compresses | 8 (44.4%) | 17 (42.5%) | 13 (33.3%) | 13 (50.0%) |
|  | Homeopathy | 3 (16.7%) | 0 | 3 (7.7%) | 1 (3.8%) |
|  | Hydrotherapy | 2 (11.1%) | 5 (12.5%) | 4 (10.3%) | 3 (11.5%) |
|  | Aromatherapy | 4 (22.2%) | 5 (12.5%) | 5 (12.8%) | 2 (7.7%) |
|  | Dietary supplements/vitamins | 10 (55.6%) | 27 (67.5%) | 19 (48.7%) | 12 (46.2%) |
|  | Prayer/spiritual healing | 3 (16.7%) | 9 (22.5%) | 17 (43.6%) | 10 (38.5%) |

CAM: Complementary and alternative medicine, HICs: High-income countries, LICs: Low-income countries, LMICs: Lower-middle-income countries, UMICs: Upper-middle-income countries
